# Association between a fish and box jellyfish during an encounter with a carcharhinid shark

**DOI:** 10.1111/jfb.70568

**Published:** 2026-07-08

**Authors:** Scott J. Morrissey, Oliver Scheele, Lawrence Scheele

**Affiliations:** ^1^ Centre for Tropical Water and Aquatic Ecosystem Research (TropWATER) James Cook University Cairns Queensland Australia; ^2^ Snorkeldownunder, Magnetic Island Yunbenun Queensland Australia

**Keywords:** associational defence, behaviour, cubozoan, fish–jellyfish interaction, predator avoidance

## Abstract

Fish‐cubomedusa associations are less frequently reported than those involving scyphozoans and are typically limited to larval or juvenile fishes without an explicit predator context. Here, we report an adult fish associating with the cubozoan *Morbakka* sp. during an encounter with a carcharhinid shark at Magnetic Island, Australia. [Correction added on 03 September 2026, after first online publication: In the previous sentence, “*Monodactylus argenteus* associated”, has been corrected to “fish associating”]. Throughout the observation, the fish maintained close association with the cubomedusa and reduced its distance to the bell and upper tentacle field during the shark encounter. Although behavioural motivations could not be determined from aerial observations alone, the observed change in spatial positioning by the fish was consistent with refuge use, although opportunistic foraging or medusivory remain plausible alternative explanations. [Correction added on 03 September 2026, after first online publication: The preceding sentence has been corrected.] This observation extends previous reports of fish‐cubomedusa associations by documenting such an interaction in an adult fish during an encounter with a carcharhinid shark.

In exposed pelagic environments, fishes may seek refuge by maintaining close proximity to jellyfish medusae, utilising them as mobile refuges in otherwise featureless environments (Mansueti, 1963). Medusae can provide fishes with structural habitat, offering refuge and foraging opportunities (Kingsford, 1993; Purcell & Arai, 2001), but may also impose constraints through competition for shared resources (Richardson et al., 2009). These associations are most commonly reported for scyphozoan jellyfish, where larval, juvenile and small‐bodied fishes are found in and around the tentacle field of medusae (Griffin et al., 2019; Purcell & Arai, 2001; Tilves et al., 2018). Such associations are generally interpreted as commensal and temporary, with fishes acting as opportunistic associates and jellyfish functioning as mobile structural hosts (Mansueti, 1963). In this context, fishes exploit the stinging capabilities of their hosts to reduce predation risk (Mansueti, 1963), and may also position themselves to capitalise on prey items entrained within the bell, tentacle field or encountered by the host (Masuda, 2008). Collectively, these fish–jellyfish associations are likely explained by the ‘meeting‐point hypothesis’ (Castro et al., 2002; Fréon & Dagorn, 2000; Soria et al., 2009), as outlined by Boco and Metillo (2018), whereby medusae act as mobile aggregation points that facilitate repeated encounters between fishes and jellyfish in otherwise featureless pelagic environments.

In contrast, associations between fishes and cubozoans remain comparatively less frequently documented, although an increasing number of observations have been reported in recent years (Corcino et al., 2025; Kingsford & Mooney, 2014; Kondo et al., 2018; Lawley & Júnior, 2018). Despite this growing body of reports, most observations remain opportunistic, limiting understanding of the frequency and broader ecological significance of these interactions. These knowledge gaps are partly attributable to the difficulty of studying cubozoans in their natural environment. Cubozoans are challenging to detect in situ and often occur patchily across space and time (Kingsford et al., 2021), which complicates their direct observation and study (Morrissey et al., 2022). Consequently, studies of cubozoan ecology often rely on indirect approaches, such as environmental sampling and modelling (Mooney & Kingsford, 2017; Morrissey et al., 2020; Morrissey et al., 2024a; Schlaefer et al., 2018), limiting the availability of direct behavioural observations (Kingsford & Mooney, 2014).

Where documented, fish‐cubomedusa interactions are typically facultative, temporary, and non‐species‐specific, and most frequently involve larval, juvenile or small‐bodied fishes (Beebe, 1928; Corcino et al., 2025; Kondo et al., 2014; Kondo et al., 2018; Lawley & Júnior, 2018). These associations are generally interpreted as antipredator behaviour, whereby fishes exploit the stinging capabilities of medusae to reduce predation risk (Kondo et al., 2018; Lawley & Júnior, 2018). However, fish‐cubomedusa interactions encompass a broader range of ecological relationships. Kondo et al. (2018) reviewed records of piscivory by cubozoans and also documented *Thamnaconus modestus* feeding on the cubozoan *Morbakka virulenta*, demonstrating that interactions between fishes and cubomedusae can be both protective and trophic in nature. Despite increasing reports of such associations, direct observations occurring in the presence of potential fish predators remain uncommon, with only isolated examples from scyphozoan systems (e.g., juvenile scads associating with scyphomedusae in the presence of predatory groupers) (Bonaldo et al., 2004). In such cases, fishes typically maintain close proximity to the medusa, often positioning themselves near or within the tentacle field.

On 29th of March 2026, at 9:17 am, a single *Morbakka* sp. medusa was observed at Horseshoe Bay, Magnetic Island, Queensland, Australia (19°06′36.9″ S, 146°51′34.9″ E). The interaction was recorded using a DJI Mini 5 Pro drone capturing high‐resolution video (4 k at 30 fps HDR video) and was continuously observable within the field of view for the majority of the recording period (~5 min). The drone was flown at an altitude of approximately 15 m using the 2 × (48 mm equivalent) zoom setting. Based on the flight altitude and camera settings, the field of view at the water surface was approximately 11.8 m wide × 6.6 m high. The approximate size of each organism was estimated from pixel measurements relative to the calculated field of view, resulting in estimated sizes of ∼30 cm total length for the fish, ∼90 cm horizontal span for the cubomedusa (including tentacles), and ∼1.3 m total length for the shark. Horseshoe Bay is the largest embayment on Magnetic Island, characterised as a shallow open coastal system, with tidal forcing driving the dominant hydrodynamic processes (Morrissey et al., 2026). The area comprises a mosaic of habitats including fringing coral reefs, seagrass meadows and sandy substrates (Brown, 1973). It is a known hotspot for some cubozoan species (Morrissey et al., 2024b), however, no other cubomedusae or large fish aggregations were observed in the immediate vicinity.

Species identification was based on morphological characteristics visible in the footage (Figure 1). The medusa was assigned to the genus *Morbakka* based on its rectangular‐cuboid bell with bell height exceeding width, pedalia length approximately half of bell height, broad and flattened tentacles with visual banding and the presence of nematocysts on the bell and pedalia (Figure 1a) (Gershwin, 2009). As some diagnostic features required for unequivocal species‐level confirmation (Bentlage & Lewis, 2012; Terenzini et al., 2023) could not be examined from aerial footage, and as regional diversity and distributions of *Morbakka* species remain unresolved, the specimen is conservatively referred to here as *Morbakka* sp. The fish was initially identified as *Monodactylus argenteus*; however, subsequent examination of the imagery indicates that this identification was incorrect. The fish could not be identified unequivocally to species from the available aerial footage. Its visible morphology, including the deep, laterally compressed body, elongate dorsal and anal fins, and forked caudal fin, is consistent with a possible black pomfret, *Parastromateus niger* (Smith‐Vaniz, 1999). However, the aerial perspective and limited visibility of diagnostic morphological features in the footage preclude confident species‐level identification. [Correction added on 03 September 2026, after first online publication: The preceding sentence has been corrected.] The shark was identified as belonging to the *Carcharhinus* genus based on a streamlined, fusiform body, a broad first dorsal fin positioned approximately above or slightly posterior to the pectoral fin insertion, moderately long and curved pectoral fins and a heterocercal caudal fin with a well‐developed upper lobe (Figure 1c). Species‐level identification was not possible due to the rippling of the water's surface which obscured several diagnostic morphological features commonly used to distinguish among carcharhinid sharks, including pigmentation patterns and markings on the caudal fin (Last & Stevens, 2009). Although the overall body shape was consistent with several carcharhinid species known from the region, including species frequently observed within Horseshoe Bay, these diagnostic features could not be resolved with sufficient confidence from the available footage. Carcharhinid sharks are known to be present or have been previously observed in the waters surrounding Magnetic Island (Scheele, 2025). [Correction added on 02 September 2026, after first online publication: In the preceding sentence, “*Monodactylus argenteus and carcharhinid sharks*” has been corrected to “Carcharhinid sharks”.] Additionally, this serves as the first reported instance of *Morbakka*'s presence in these waters. Observations were conducted non‐invasively using aerial video footage.

**FIGURE 1:**
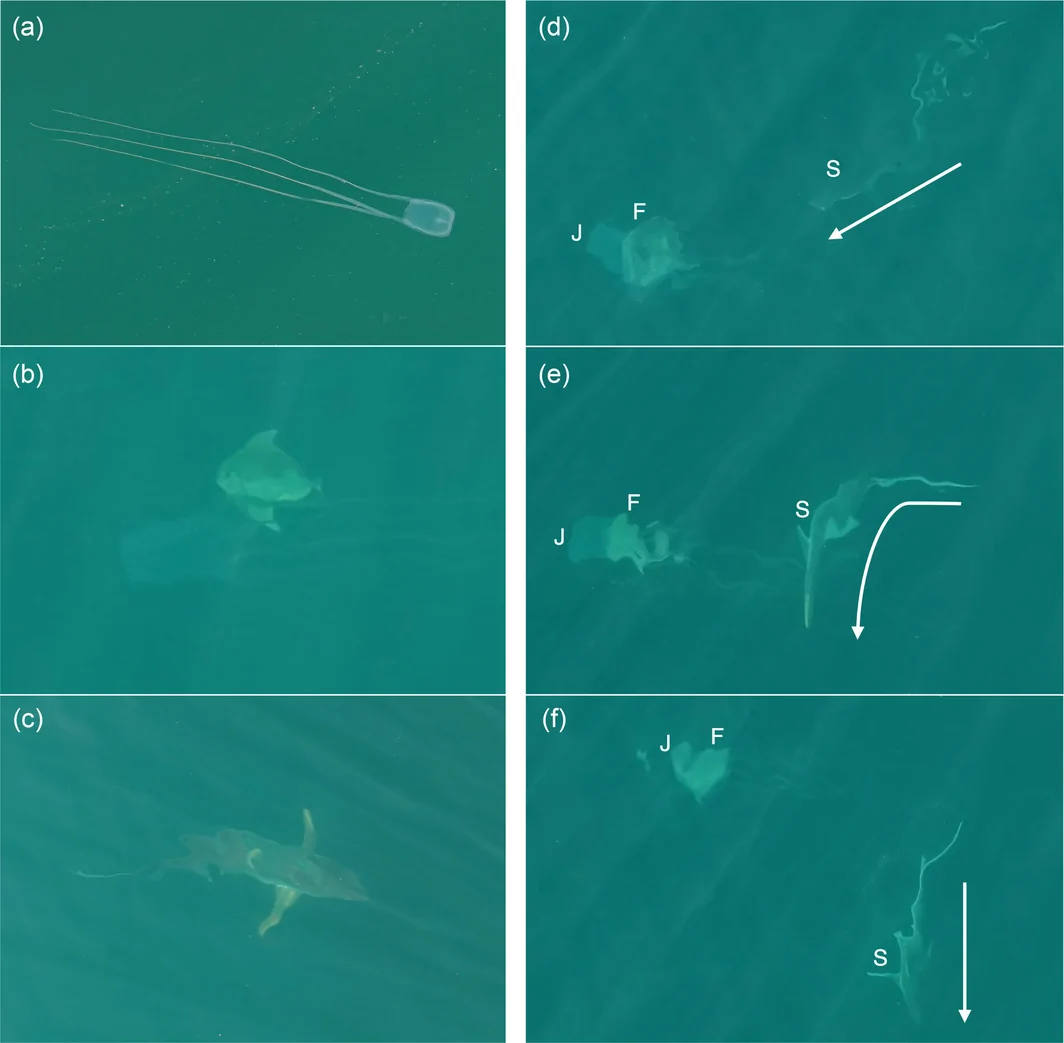
Representative frames used for taxonomic assessment (a) Morbakka sp. showing characteristic cuboid bell and trailing tentacles; (b) fish, possibly *Parastromateus niger*, displaying a deep, laterally compressed body, elongate dorsal and anal fins, and forked caudal fin; and (c) *Carcharhinus* sp. showing body shape and fin configuration consistent with the genus. Sequential frames illustrating the interaction between *Morbakka* sp., the fish and *Carcharhinus* sp. (d) *Carcharhinus* sp. approaching the medusa‐fish pair; (e) *Carcharhinus* sp. at closest proximity, adjacent to the tentacle field and altering trajectory; (f) *Carcharhinus* sp. departing without entering the immediate vicinity of the tentacles. White arrows are indicative of *Carcharhinus* sp. direction and movement. Labels J, F and S are indicative of jellyfish, fish and shark locations, respectively. The fish remained in close proximity to the medusae throughout the interaction.

An adult‐sized fish was observed maintaining close association with the cubomedusa, typically positioning below and slightly posterior to the bell (Figure 1d). [Correction added on 03 September 2026, after first online publication: In the preceding sentence, “*M. argenteus*” has been corrected to “fish”.] The fish adjusted its position in response to medusa movement and remained closely aligned throughout the observation. During the observation, a *Carcharhinus* sp. circled the medusa‐fish pair before turning toward the pair and making its closest approach (Figure 1e, Video S1). The shark maintained a steady cruising speed throughout the encounter, with no apparent acceleration. Throughout the encounter, the fish maintained a reduced distance to the medusa, remaining within close proximity to the bell and upper tentacle field. The shark subsequently altered its trajectory and departed without entering the immediate vicinity of the tentacles (Figure 1e,f). No predation attempt or direct contact was observed.

During the shark encounter, the apparent distance between the fish and cubomedusa decreased in the aerial footage. This pattern is consistent with a possible behavioural response to the shark's presence (e.g., seeking shelter near the cubomedusa). Fine‐scale positioning, particularly below and adjacent to the bell, may allow fish to maximise protection while minimising contact with tentacles, as has been described in fish‐scyphozoan associations (Brodeur, 1998). If the fish deliberately reduced its distance to the cubomedusa in response to the shark, the interaction would be consistent with facultative associational defence, whereby fishes exploit the stinging capabilities of medusae to reduce predation risk (Mansueti, 1963). [Correction added on 03 September 2026, after first online publication: The preceding sentence has been corrected.] In this context, cubomedusae may function as temporary mobile refuges, providing shelter in otherwise open or low‐structure environments where conventional refuges are limited or absent. However, alternative explanations for the association cannot be excluded. The fish may have remained associated with the medusa for opportunistic foraging or potentially direct feeding, independent of the shark's presence, although no feeding behaviour was directly observed here. Consequently, while the apparent reduction in distance between the fish and cubomedusa during the shark encounter is consistent with predator avoidance, the behavioural motivation for the association remains uncertain. These explanations are not mutually exclusive, and multiple behavioural drivers may have operated simultaneously. [Correction added on 03 September 2026, after first online publication: The preceding sentence has been corrected.] Notably, the interaction involved an adult fish and occurred during an encounter with a carcharhinid shark, providing a rare observational example of a fish‐cubomedusa association in the presence of a potential predator.

Interpretation of fine‐scale spatial relationships among the fish, cubomedusa, and shark is constrained by the aerial perspective of the footage. As observations were derived from a top‐down view, vertical separation among organisms could not be quantified and true three‐dimensional distances may differ from those apparent in the video. Similarly, surface glare and optical distortion may have influenced estimates of organism size and spacing. While aerial platforms provide valuable opportunities to observe marine fauna and behaviour (Hodgson et al., 2013; Rieucau et al., 2018), complementary underwater observations can provide important additional information on animal positioning and behaviour within the water column. Consequently, behavioural interpretations should be regarded as suggestive rather than definitive. Future studies combining aerial observations with underwater video or stereo‐imaging systems would provide greater resolution of these interactions and allow fine‐scale behavioural responses to be quantified more accurately.

Associations between fishes and jellyfish are well documented for scyphozoans, whereas reports involving cubozoans are comparatively less common (Lawley & Júnior, 2018). Existing records predominantly involve juvenile or small‐bodied fishes, with associations typically interpreted as temporary, antipredator strategies (Corcino et al., 2025; Kondo et al., 2018; Lawley & Júnior, 2018). Moreover, although several studies have documented trophic interactions involving fishes and cubomedusae, including medusivory (Kondo et al., 2018), few observations have explicitly documented fish‐cubomedusa associations occurring in the presence of potential predators of the associated fish, limiting understanding of the functional role of these associations during such encounters. Unlike most previous reports, the present observation involved an adult fish and occurred during an encounter with a carcharhinid shark, with the fish maintaining a close association with the cubomedusa throughout the encounter. Although this association is consistent with possible refuge use, alternative explanations, including opportunistic foraging or direct feeding, cannot be excluded. [Correction added on 03 September 2026, after first online publication: The preceding sentence has been corrected.] This observation suggests that such associations are not restricted to early life stages, but may instead represent a flexible, context‐dependent strategy employed by fishes when potential predators are present. By documenting an adult fish maintaining close association with a cubomedusa during an encounter with a carcharhinid shark, this observation expands the range of ecological context in which fish‐cubomedusa associations have been reported. This account is, however, based on a single opportunistic observation, and further observations are required to determine the frequency and ecological significance of such interactions.

## AUTHOR CONTRIBUTIONS

**Scott J. Morrissey:** Conceptualisation; data curation; formal analysis; writing—original draft; writing—review and editing. **Oliver Scheele:** Conceptualisation; data curation; formal analysis; data collection; writing—review and editing. **Lawrence Scheele:** Conceptualisation; data curation; formal analysis; data collection; writing—review and editing.

## CONFLICT OF INTEREST STATEMENT

The authors declare no conflicts of interest.

[Correction added on 03 September 2026, after first online publication: In the reference section, Froese et al. has been removed and Smith‐Vaniz has been added.]

## Supporting information


**Video S1.** Drone footage showing an adult *Monodactylus argenteus* maintaining close association with a *Morbakka* sp. medusa during an encounter with a *Carcharhinus* sp. in Horseshoe Bay, Magnetic Island, Queensland, Australia.

## Data Availability

The data that support the findings of this study are available from the corresponding author upon reasonable request.
